# Pathological bases and clinical impact of long noncoding RNAs in prostate cancer: a new budding star

**DOI:** 10.1186/s12943-018-0852-7

**Published:** 2018-07-23

**Authors:** Tao Xu, Chang-ming Lin, Shu-qi Cheng, Jie Min, Li Li, Xiao-ming Meng, Cheng Huang, Lei Zhang, Zi-yu Deng, Jun Li

**Affiliations:** 1 School of Pharmacy, Anhui Key Laboratory of Bioactivity of Natural Products, Anhui Medical University, 81 Meishan Road, Hefei, 230032 China; 2 Institute for Liver Diseases of Anhui Medical University, Anhui Medical University, Hefei, 230032 China; 3 Department of Urology, The Fourth Affiliated Hospital of Anhui Medical University, Hefei, 230022 China; 4 Department of Urology, The Second Affiliated Hospital of Anhui Medical University, Hefei, 230601 China; 5 Department of Pathology and Pathophysiology, Zhejiang University School of Medicine, Hangzhou, China; 6 Department of Scientific, The Second Affiliated Hospital of Anhui Medical University, Hefei, 230601 China

**Keywords:** Long non-coding RNAs, Prostate cancer (PC), Biomarker, Tumorigenesis

## Abstract

Long non-coding RNAs (lncRNAs) are functional RNAs longer than 200 nucleotides. Recent advances in the non-protein coding part of human genome analysis have discovered extensive transcription of large RNA transcripts that lack coding protein function, termed non-coding RNA (ncRNA). It is becoming evident that lncRNAs may be an important class of pervasive genes involved in carcinogenesis and metastasis. However, the biological and molecular mechanisms of lncRNAs in diverse diseases are not yet fully understood. Thus, it is anticipated that more efforts should be made to clarify the lncRNA world. Moreover, accumulating evidence has demonstrated that many lncRNAs are dysregulated in prostate cancer (PC) and closely related to tumorigenesis, metastasis, and prognosis or diagnosis. In this review, we will briefly outline the regulation and functional role of lncRNAs in PC. Finally, we discussed the potential of lncRNAs as prospective novel targets in PC treatment and biomarkers for PC diagnosis.

## Highlight

1. The oncogenic or tumor suppressive roles of lncRNA in PC.
2. The epigenetical silencing role of lncRNA in PC.
3. The epithelial–mesenchymal transition role of lncRNA in PC.
4. Potential clinical application of lncRNA as diagnostic marker in PC.

## Background

Prostate cancer (PC) is the most common malignancy in male and accounts for 13% of cancer-related deaths [1]. It is reported that the 5-year survival rate of PC patients was only 29%, exerting a big burden on public health a worldwide [2]. The latest data indicate that the incidence and mortality of PC are estimated to reach up to 180,890 new cases and 85,920 new deaths by the year 2016 in the US. According to the Cancer Statistics report, there will be an estimated 161,360 new cases diagnosed and 26,730 deaths in 2017 [3]. Moreover, increased detection of PC is attributed to prostate-specific antigen screening and improved biopsy techniques, early-stage PC patients often show good prognosis after comprehensive treatment. Nevertheless, recurrence, metastasis and development of castration-resistant PC remain the leading causes of mortality [4]. Therefore, the discovery of new molecular targets for PC may help to understand the pathogenesis and prognosis of PC.

The ENCODE (encyclopedia of DNA elements) project showed that the vast majority (80.4%) of the human genome participates in at least one biochemical RNA and/or chromatin-associated event in cells [5]. Nevertheless, only 2% of the genome sequences encode proteins, the remaining is transcribed into noncoding RNAs (ncRNAs) [6]. To date, lncRNAs (> 200 nt), well-known discovered ncRNAs, with limited or no protein-coding capacity, serve primarily as the regulatory components in the cell and they are reported as implicated in a vast number of cellular processes. Dysregulation of lncRNA in various cancers is considered as one of the leading forces during tumorigenesis by influencing tumor cell proliferation, evading growth suppressors, enabling replicative immortality, enabling replicative immortality, inducing angiogenesis and resisting cell death, etc. [7, 8]. Most recently, many studies had proved that lncRNAs played an important role in the carcinogenesis of cancer, especially in PC. Notably, HOTAIR (HOX transcription antisense RNA) was reported to be up-regulated in PC, and HOTAIR silencing significantly suppressed the proliferation and migration of PC [9]. Another study revealed that lncRNA LOC400891 acted as a novel prognostic biomarker and therapeutic target of PC [10]. Therefore, in this review, we will briefly outline the biogenesis of lncRNA, highlight the functional roles of several lncRNAs in PC and discuss the application of lncRNA as a target for PC treatment and a biomarker for PC diagnosis.

### Overview of lncRNA

LncRNA is generally defined as an RNA molecule and lacks coding potential [11]. LncRNAs are roughly divided into five categories according to their localization on the genome. In addition, according to their special features, lncRNAs can be segmented into lncRNA activating (lncRNA-a) genes, pseudogenes, telomere-associated ncRNAs (TERRAs), transcribed ultraconserved regions (T-UCRs), enhancer RNAs (eRNAs), circular RNAs and so on [12]. lncRNAs seem to be transcribed similarly to mRNAs and they can regulate gene expression directly in adjacent genomic loci (cis) or by initially interacting with transcriptional regulators (trans) [13]. The current release GENCODE (Encyclopædia of genes and gene variants) Version 24 (V24) released in 2015 for humans has in total 60,554 genes annotated as protein-coding genes (19,815), lncRNA genes (15,941) and small ncRNA genes (9882). It is also one of the most comprehensive annotations for lncRNAs genes [14]. Works on the structure of lncRNAs indicated that functional lncRNAs share some primary sequence features, including paucity of introns, low PC content, poor start codon and open reading frame contexts [15]. Noteworthily, the presence of motifs embedded in the lncRNA primary sequence endows lncRNAs with the ability to bind DNA, RNA and/or protein and thus highly correlated with diseases [16]. Recently, genome-scale approaches were developed to measure RNA secondary structures. As a result, many lncRNAs have a significant secondary structure, which is critical for specific binding and function [17]. For instance, HOTAIR can form multiple double stem–loop structures for binding to the lysine-specific demethylase 1 and polycomb repressive complex 2 (PRC2) histone modification complexes [9]. PRC2 is among the main chromatin regulatory factors and is known for its ability to regulate epigenetic cellular memory, cancer development through tri-methylation of specific lysine (K) residues of K9 and K27 in histone H3 [18]. The complicated structure enables lncRNAs with several regulatory capacities. They act as decoys, activators, guides or scaffolds for their interacting proteins.

LncRNAs have been implicated in multiple processes, including chromosome dosage compensation, control of cell proliferation, cell differentiation, apoptosis, regulation of epithelial-mesenchymal transition (EMT) and nuclear and cytoplasmic trafficking [19]. Recent studies have indicated that lncRNAs can mediate a “sponge” regulatory network (sequestering microRNAs) that can differentially affect the expression of many protein-coding PC driver genes and key components of cancer-driving pathways during carcinogenesis [20]. Moreover, some lncRNAs are linked to reactivation of the androgen receptor signaling axis and reprogramming of PC cellular metabolism, and thus may be differentially expressed during various phases of tumor development and progression [21]. Considering their dynamic role in PC, lncRNAs may also serve as therapeutic targets, helping to prevent development of castration resistance, maintain stable disease and prohibit metastatic spread.

### Roles of LncRNAs in PC

The carcinogenesis of PC is complex and includes multiple processing steps, involving numerous genetic and epigenetic alterations [22]. Although the functions of lncRNA are gradually be understood, dysregulation of lncRNAs is becoming a ubiquitous component in the gene regulatory networks of cancer development and progression [23]. Several studies have showed that down-regulation or up-regulation of lncRNA expression attributes a tumor-suppressor or an oncogenic role to lncRNAs affecting the clinicopathological appearance, prognosis and outcome of PC. It’s worth mentioning that the role of these lncRNAs in PC is reviewed in greater detail (Table 1).

**Table 1:** The expression of LncRNA in PC

LncRNA	Cytology	Location	Expression	Function in tumorigenesis	Reference(PMID)
PCGEM1	2q32.2	PCa Tissues,LNCaP NIH3T3 Nuclear	+	Oncogene	11050243, 14724589, 27976428
PCAT1	8q24.2	PCa Tissues, DU145 Cytoplasma	+	Oncogene	24473064
MALAT1	11q13.1	PCa Tissues, Urinary	+	Oncogene/Diagnosis and Prognosis marker	23845456, 25526020
PVT1	8q24.21	PCa Tissues	+	Oncogene	27794184
GAS5	1q25.1	PCa Tissues	–	Tumor suppressor	27743383
CTBP1-AS	4p16.3	VCaP Nucleus	+	Oncogene	23644382
PCA3	9q21.22	Peripheral Blood	+	Diagnosis marker	27743381
SChLAP1	2q31.3	PCa Tissues	+	Metastasis/Prognosis marker	25499224
LINC00963	9q34.11	LNCap cells	+	Metastasis	24691949
SOCS2-AS1	12q22	PCa Tissues	+	Oncogene	28241429, 27342770
lncRNA FR0348383	–	PCa Tissues	+	Diagnosis and Prognosis marker	28272371
lnc-MX1–1	21q22.3	PCa Tissues	+	Prognosis marker	26797783, 26797523
PCAT-14	22q11.23	22Rv1 VCaP Nucleus, Plasma	+	Prognosis marker	27460352, 27566105
lincRNA-p21	upstream of CDKN1A	Urine Exosomes	–	Tumor suppressor/Prognosis marker	28272371, 27976420, 25999983
HCG11	6q22.2	PCa Tissues	–	Prognosis marker	27522256
LncRNA-ATB	14	PCa Tissues	+	Treatment target/Prognosis marker	27176634
UCA1	19p13.12	PCa Tissues	+	Prognosis marker	27902466
NEAT1	11q13.1	PCa Tissues	+	Prognosis/Predictive marker	28241429
DRAIC	15q23	LNCap Cytoplasma	–	Tumor suppressor	26200579
Lncrna SNHG1	–	PCa Tissues	+	Oncogene	28400279
Lncrna HOXD-AS1	2q31.1	PCa Tissues	+	Oncogene	28487115
CCAT-2	8q24.22	PCa Tissues	+	Prognosis marker	27558961
HOTAIR	12q13.13	PCa Tissues, CRPC cell Cytoplasma	+	Oncogene/Prognosis marker	26411689
MEG3	14q32.3	PCa Tissues	–	Tumor suppressor	26610246, 28241429
H19	11p15.5	M12 Cytoplasma	–	Tumor suppressor	24988946
PCAT-18	18q11.2	PCa Tissues, Plasma	+	Diagnosis marker/Treatment target	24519926, 28272371
DANCR	4q12	PCa Tissues	+	Metastasis	27191265
PCAT-29	15q23	LNCap Nucleus	–	Tumor suppressor	25200579
LINC01296	14q11.2	PC cell Nucleus, Cytoplasma	+	Metastasis/Treatment target/Prognosis marker	28392705, 28341852
POTEF-AS1	2q21.2	PCa Tissues	+	Oncogene	28032932
GLIDR	9p12	PCa Tissues	+	Oncogene	28211799
PCAT-5	10p11.21	PCa Tissues	+	Oncogene	26282172
THBS4–003	5p14.1	PCa Tissues	+	Metastasis	27357608
lncRNA LOC400891	–	PCa Tissues	+	Prognosis marker	26797783

Upregulation:+; Downregulaition: -

### The oncogenic or tumor suppressive roles of lncRNA

PC gene expression marker 1 (PCGEM1), located on chromosome 2q32.2, is over-expressed in 84% PC patients by in situ hybridization [24]. PCGEM1 over-expression in PC-derived LNCaP cells promotes proliferation and a dramatic increase in colony formation [25]. Similarly, according to Petrovics’s report, over-expression of PCGEM1 evidently enhanced LNCaP cell proliferation and colony-forming capacity. In aspect of cell cycle related genes, the biological function of PCGEM1 was resulted from its ability to stimulate Rb (Retinoblastoma) protein phosphorylation at serine 801 residue, which prevented Rb from binding to E2F and allowed cell to enter into S phase. Importantly, ChIP-3C assay confirmed that PCGEM1 make the distant regulation of AR (Androgen Receptor) by exerting as scaffold lncRNA to connect chromatin structure together, then bring Rb together to let the phosphorylation reaction happen [26]. Moreover, Yang et al. reported PCGEM1 is highly over expressed in aggressive PC and bind successively to the AR protein to enhance the AR-mediated gene activation program and induce PC growth [27]. Hung et al. also revealed PCGEM1’s critical role in metabolic regulation independent of androgen and AR. They found that PCGEM1 directly bound c-Myc, which is a ubiquitous transcription factor essential for cell cycle progression, enhanced c-Myc transactivation potency [28]. Moreover, Hung et al. showed that the structural domain for c-Myc binding was consistent as the functional domain for c-Myc target-gene regulation. Thus, PCGEM1 can provide growth advantages for PC cells by regulating tumor metabolism via c-Myc activation [28]. In addition, with the support of new technology of CRISPR/Cas9 technology (a dual gRNA approach), Ho et al. have confirmed that PCGEM1 is functionally regulated by p54/nrb, which is a multi-functional nuclear protein involved in a variety of nuclear processes, especially playing a role in RNA splicing and gene regulation [29].

Differentiation antagonising non-protein coding RNA (DANCR), located on chromosome 4q12, suppresses differentiation of epithelial cells [30]. Jia et al.found that the DANCR level in PC specimens are much higher than that in adjacent normal tissues and the up-regulated DANCR promotes invasion and migration of PC cells in vitro and metastasis of tumor xenografts in nude mice [18]. Furthermore, TIMP2/3, which are critical metastasis inhibitor of PC, were down-regulated by DANCR synergistically with EZH2 (Enhancer of zeste homolog) through epigenetically silencing their promoter. EZH2 is the catalytic part of the PRC2 which catalyzes the trimethylation of Histone 3 on lysine 27 (H3K27me3) and induces chromatin compaction and transcription repression [31] and DANCR could bind to EZH2 [32]. It was previously documented that AR is an inhibitor of PC metastasis. DANCR was down-regulated by androgen-AR signaling pathway in PC. Importantly, AR knockdown also decreased TIMP2/3 expression, thereby contributing to PC cells invasion and migration [18]. Thus, collectively, DANCR impedes the upregulation of TIMP2/3 and the suppression of invasion and migration by androgen-AR signaling pathway.

PC-associated intergenic non-coding RNA transcript 1 (PCAT1)exhibits frequent chromosomal amplification resides in chromosome 8q24 [33, 34]. In PC patients, PCAT1 is overexpressed in tissues and it promotes prostate cells of Du145 and RWPE proliferation. Prensner JR et al. confirmed that PCAT1 mediated proliferation is dependent on c-Myc protein stabilization and c-Myc is required for a subset of PCAT1-induced expression changes [35]. Additionally, Ma et al. show that the expression of PCAT1 is mutually exclusive to that of EZH2, which is a component of PRC2. Indeed, PRC2 was recruited to the promoter of PCAT1 by ChIP assay [34]. Recently, rs7463708, a PC risk-associated SNP located 78 kb downstream of the PCAT1 transcription start site (TSS), modulated the activity of PCAT1 enhancer, resulting in an increased PCAT1 expression and consequently [36].

LINC01296, located at chromosome 14q11.2, was significantly up-regulated in PC cell lines (22Rv1 and LNCaP) and PC tissues [4]. A recent study demonstrated that LINC01296 can exhibit oncogenic properties by promoting proliferation, migration and invasion in bladder cancer [37]. Soon after, Wu et al. found the LINC01296 had the same function in PC. LINC01296 knockdown inhibited the proliferation, migration and invasion of 22Rv1 and LNCaP cell. The PI3K/Akt/mTOR signaling pathway, one of the three main signaling pathways, plays an important role in the development and progression of PC [4, 38]. Interestingly, LINC01296 knockdown significantly reduced the expression levels of phosphorylated PI3k (p-PI3k), phosphorylated Akt (Ser473)(p-Akt) and phosphorylated mTOR (p-mTOR). This result suggested that LINC01296 is involved in the promotion of PC cells proliferation and seems to be mediated by regulation of PI3K/Akt/mTOR pathway [4]. Consistent with the finding is that this pathway is also applied to the POTEF-AS1. POTEF-AS1 promotes PC cell lines (LNCaP and LTAD) growth by targeting TNFSF10 (the tumor necrosis factor superfamily member 10) which belongs to pro-apoptotic protein ligands of the tumor necrosis factor superfamily and TLR3 which triggers apoptosis and growth arrest of LNCaP cells partially through the inactivation of the PI3K/Akt signaling pathway [39].

HOTAIR, a ~ 2.2 kb lncRNA which was identified from the trimethylate histon H3 lysin-27 of HOXD locus on chromosome 12, was firstly found to have trans transcription regulation effect and ever described to interact with PRC2 and repress transcription in trans of HOXD in foreskin fibroblasts [40]. HOTAIR has been recognized to be strongly associated with metastasis in various cancers, including breast cancer, renal cell carcinoma, colorectal cancer and PC [34]. In PC, HOTAIR, an androgen-repressed lncRNA, is up-regulated in androgen independent cell line C4-2B. HOTAIR is markedly upregulated following androgen deprivation therapies and in CRPC (castration resistant PC). In addition, its expression is sufficient to induce androgen-independent AR activation and drive the AR-mediated transcriptional program in the absence of androgen. Targeted expression of HOTAIR in LNCaP could promote cell growth and cell invasion even in the absence of androgen stimulation. Meanwhile, knockdown of HOTAIR in C4-2B cell line dramatically suppressed cell proliferation and cell invasion, indicating HOTAIR was tumor-progression driver in CRPC and increased PC cell growth and invasion. In the previous study, HOTAIR can simultaneously bind to the EZH2 and LSD1 (Lysine Specific Demethylase 1) proteins through its 5′ end and 3′ end, respectively, direct these proteins to co-occupy the same genomic regions as a scaffold. Through this scaffold function, HOTAIR also can link E3 ubiquitin ligases with their substrates to accelerate proteolysis [9]. Taken together, HOTAIR plays an oncogenic role to promote PC cell proliferation and invasion.

LncRNA SChLAP1 (second chromosome locus associated with prostate-1, LINC00913), was first identified by John R Prensner et al. and located in the nucleus [41]. Elevated SChLAP1-levels are even more frequent in mCRPC(metastatic CRPC) [42]. In addition, the expressions of SChLAP1 in PC tissues and four PC cell lines (LNCap, 22Rv1, DU145, PC-3) were significantly higher than in normal tissues and RWPE-1 cells. The expression levels of Ki67 and PCNA were obviously decreased in the SChLAP1-siRNA transfected LNCaP and PC-3 cells [42]. Meanwhile, the percentage of Annexin V positive apoptotic cells were increase, accompanied by increased expression of Caspase-3 and Caspase-9. In addition, the upregulated SChLAP1 promoted the migration and invasion of PC cells by promoting the expression of VEGF (vascular endothelial growth factor) and MMPs (matrix metalloproteinases) [42]. Elevated levels of MMP-2 and MMP-9 are observed in prostate cancer and correlate with increased metastasis [43]. VEGF could generate a spatial gradient of endothelial cell proliferation and angiogenesis toward ischamic areas, permitting cell proliferation and tumor expansion [44]. Moreover, it reported that SChLAP1 can interact with the SWItch/Sucrse Non-Fermentable (SWI/SNF)-complex. Any loss of this complex moves nucleosomes at gene promoters, resulting in cancer progression. At the post-transcriptional level, SChLAP1 counteracts tumor suppressive effects of the SWI/SNF complex by impairing its capability to regulate gene expression [42]. Taken together, the upregulated SChLAP1 promoted proliferation, migration, invasion and promote apoptosis of PC cells.

Metastasis-associated lung adenocarcinoma transcript 1 (MALAT-1), a noncoding RNA of more than 8000 nt derived from chromosome 11q13, has been described as a regulator of metastasis and motility [45]. It was well documented as critical regulator in metastasis, alternative RNA splicing, nuclear organization and epigenetic modification [46]. In 2013, Sun and colleagues demonstrated that MALAT1 was significantly elevated in human PC. MALAT1 silencing reduced cell growth and metastasis in PC cells. In vivo xenograft animal model, delivery of siRNA against MALAT1 contributed to the delay of tumor growth and inhibition of metastasis. Therefore, these results implied that MALAT1 may function as an onco-lncRNA in PC [47].

The lncRNA CTBP1-AS (C-terminal binding protein 1-antisense) is located in the antisense region of the CTBP1 gene. It is predominantly localized in the nucleusand and its expression is generally upregulated in PC. Takayama et al. ‘s data show that CTBP1 functions as a AR corepressor by inhibiting androgen-mediated demethylation and CTBP1 overexpression reduced PC cell proliferation with accompanying repression of androgen-regulated genes and that knocking down CTBP1 increased LNCaP cell proliferation. These results demonstrated the importance of CTBP1 in controlling cancer proliferation. In addition, CTBP1-AS irepresses transcription of CTBP1 by recruiting histone decarboxylase (HDAC)-paired amphipathic helix protein Sin3A complexes to the gene’s promoter region, after having associated with the transcriptional repressor PTB (Polypyrimidine Tract Binding Protein)-associating splicing factor (PSF). Thus, high levels of CTBP1-AS are accompanied by reduced CTBP1 levels. Moreover, PSF and CTBP1-AS promote cell cycle progression by repressing Mothers against decapentaplegic homolog 3 (SMAD3) and p53, two cell cycle inhibitors usually regulated by the AR. Additionally, CTBP1-AS itself mimics AR signalling by inducing upregulation of AR-regulated genes. During androgen deprivation therapy, expression levels of CTBP1-AS constantly increase. Especially in androgen-deprived conditions, the lncRNA promotes cell cycle progression and tumour cell proliferation. During treatment with anti-androgens, tumor cell proliferation may be reduced via targeting CTBP1-AS and its protein partner PSF [48].

Maternally Expressed Gene 3 (MEG3), located on chromosome 14q32, is an imprinted gene expressed from the maternal allele with a length of about 1.6 kb nucleotides [49]. Luo et al. found the MEG3 expression was significantly downregulated in PC tissues. Overexpression of MEG3 increased the proportion of cells in G0/G1 phase and decreased the proportion of cells in S phase [49]. In the aspect of mechanism, MEG3 normally acts as a tumour suppressor, as it likewise impairs cell proliferation and promotes apoptosis by activating p53 [50]. p53 plays a tumor suppression role in most human cancers [51]. Additionally, Luo et al. confirmed that MEG3 inhibited intrinsic cell survival pathway by reducing the protein expression of Bcl-2(B-cell lymphoma-like 2) and cyclin D1, enhancing Bax and activating caspase-3. The Bcl-2 protein plays an important role in preventing cancer cell apoptosis and Bax is known for its pro-apoptotic activity.Caspase-3 which can be observed to be activated by MEG3 is the key mediator in the apoptotic pathway [49].

Long intragenic non-coding RNA lincRNA-p21, a transcript expressedfroma locus between CDKN1A and SFSR3, is found in exosomes. Exosomes are membranous vesicles containing various biomolecules including lncRNAs, which are involved in cellular communication and are secreted from many cells including cancer cells. lincRNA-p21 was ~ 3.0 kb in length, like the mouse counterpart [52]. The physiological and pathological functions of lincRNA-p21 were gradually defined. For example, lincRNA-p21 has been identified as a regulator for the War-burg effect and as a valuable therapeutic target for cancer [53]. lincRNA-p21 level was significantly down-regulated in PC tissues. lincRNA-p21 inhibits PC cell growth and induces cell apoptosis in vitro and in vivo. The down-regulated lincRNA-p21 can promote PC cell proliferation [54]. Interestingly, Huarte M et al. performed nuclear fractionation experiments and confirmed that lincRNA-p21 is also enriched in the nucleus. Huarte M et al.’s observation demonstrated that lincRNA-p21 played an important role in the p53-dependent induction of cell death. Moreover, they further demonstrated that lincRNA-p21 bound to hnRNP-K (heterogeneous nuclear ribonucleoprotein K) [55], which is a component of a repressor complex that acts in the p53 pathway [56]. Collectively, the mechanism of lincrna-p21 mediated transcriptional repression in the p53-dependent induction of cell death is through interaction with hnRNP-K. Consistent with Huarte M, Wu et al. also found lincRNA-p21 could modulate the function of p53 in regulating cell proliferation and apoptosis [57]. Furthermore, Wang et al. found lincRNA-p21 promoted the expression of p53 downstream Mdm2, Puma, Noxa and Bax. The result indicated that lincRNA-p21 may regulate p53 enrichment at the promoters of Mdm2, Puma, Noxa and Bax to mediate their expression and apoptosis [54]. In summary, lincRNA-p21 serves as a tumor suppressor in human PC by regulating p53 and its downstream genes.

DRAIC(down-regulated RNA in androgen independent cells, LOC145837) and PCAT29 (PC-associated transcript 29), both locus on human chromosome 15q23, were reported as novel tumor suppressive gene in 2015 [58]. The PCAT29-locus is situated 20 kb downstream to DRAIC [58]. DRAIC is a spliced transcript of 1.7 kb that is expressed mainly in the cytoplasm and PCAT29 is a spliced transcript of 0.69 kb that is expressed mainly in the nuclear. In the PC patients, the expression of DRAIC and PCAT29 is down regulated in C4-2B cells and other CR (castration resistant) cells compared to LNCaP cells [59]. The expression of lncRNA PCAT29 is repressed by AR. According to the functional analysis, DRAIC prevents the transformation of cuboidal epithelial cells to fibroblast-like morphology and prevents cellular migration and invasion. However, knockdown of DRAIC represses cell proliferation while knockdown of PCAT29 induces proliferation [60]. In addition, both of DRAIC and PCAT29 are regulated by the androgen–AR-axis. The AR is recruited both to the DRAIC and PCAT29 gene loci and represses their transcription. It is interesting that FOXA1 and NKX3–1 could occupy the same regions where AR is recruited at DRAIC promoter. FOXA1 (the transcription factors forkhead box protein A1) and NKX3–1 (homeobox proteinNKX3–1) are positive transcription factors for DRAIC and PCAT29, both of which decrease migration and invasion and predict good prognosis. Thus, they can counteract the suppressive effect of AR on PCAT29 and DRAIC gene loci by inducing their transcription [58].

GAS5 (Growth arrest-specific 5), located on chromosome 1q25.1, expressed a relatively low level of PC3, DU145, and LNCaP cells in comparison to RWPE-1 normal prostate cells [61] and its reintroduction accelerates apoptosis in prostate cancer cell lines [62]. William et al. reported the molecular requirements for the recognition of steroid receptors (SRs) by the lincRNA GAS5, which regulates steroid-mediated transcriptional regulation, growth arrest, and apoptosis. GAS5 could inhibit the transcriptional activity of SRs through direct competition for DNA binding, this repression is mediated through sequence-specific protein RNA-contacts within an A-form double helical structure with a widened major groove that facilitates SR binding and resulting in repression of steroid-mediated transcription [62]. In addition, some literatures reported that the mutation of lncRNA site also affected the life activity of PC cells. Hudson et al. identified G549A mutation on GAS5 is sufficient to ablate the interaction between SRs and GAS5 both in vitro and in cells and the GAS5 G549A mutant prevents GAS5-induced apoptosis in 22Rv1 PC cells. The lncRNA GAS5 could regulate steroid-mediated transcriptional regulation, growth arrest, and apoptosis. Consequently, in steroid-driven cancer cells such as 22Rv1 cells, GAS5 function appears to be directly related to the SR-GAS5 signaling axis [62].

In addition to the common long chain RNA that affects the growth of PC cells, there are other lncRNA that also affect its growth. For instance, over-expression of SOCS2-AS1(The lncRNA Suppressor of cytokine signalling 2-antisense transcript 1) enhanced proliferation and migration of PC cells and it inhibits PC cells apoptosis by down regulating the expression of TNFSF10, particularly in castration-resistant cell lines [50, 63]. CCK8 assay showed that inhibition of UCA1 (Human urothelial carcinoma associated 1), which located in chromosome 19p13.12, suppressed PC cell proliferation, migration and invasion [64]. And H19 was significantly down-regulated in metastatic prostate cell M12 compared with P69 cell line and over-expression of H19 significantly inhibited PC3 cells migration [65].

Gene fusions are common features of neoplasia. Qin et al.found the fusion RNA, SLC45A3-ELK4, a fusion transcript functions as a long non-coding chimeric RNA (lnccRNA), was generated by cis-splicing between neighboring gene in PC and encoded the same protein as ELK4. Silencing the fusion transcript, but not wild type ELK4 resulted in slower cell proliferation in both LNCaP and PC3 cells. Intriguingly, a mutant fusion RNA, a TGG- > TGA modification located 34 nucleotides after the fusion junction site, that fails to translate into ELK4 protein rescued growth arrest caused by the fusion RNA silencing. In addition, the mutant fusion RNA re-suppressed the downstream targets of SLC45A3-ELK4 in both cases done in LNCaP and PC-3 cell. Taken together, these results demonstrated that the non-coding function of the fusion regulated PC cell proliferation and it provided a new direction for the study of prostate cancer in the field of lncRNA fusion [66].

### The role of LncRNA in epigenetical silencing

LncRNAs have emerged as important molecular players in the regulation of gene expression in different biological processes. They are involved in epigenetic processes, leading to the establishment of chromatin conformation patterns that ultimately result in the fine control of genes [67]. Epigenetic mechanisms have been involved in the differentiation of many cell types from progenitor or primary cells and with whom they share the same DNA sequence [68]. The major epigenetic features of PC cells include DNA methylation, posttranslational histone modifications and RNA-based mechanisms including those controlled by micro RNAs (Table 2).

**Table 2:** The targets of LncRNA in PC

LncRNA	Target	PC cells	Reference(PMID)
PCEGM1	miR-145	LNCaP,PC3,DU145	25200485
H19	miR-675	P69, PC3, M12	24988946
HOTAIR	miR-331-3p, PCR2	LNCaP,LAPC4	27794184
LncRNA PVT1	miR-146a	LNCaP,PC3,DU145	27794184
LncRNA GAS5	miR-103	LNCaP,PC3,DU145	27743383
PCAT-1	miR-34a	LNCaP,DU147	25425964
HOXD-AS1	WDR5 H3K4me3	PC3	28487115
UCA1	miR-204, ATF2	LNCaP,PC3,DU145	28337266
CTBP1-AS	p53, CTD3BP1, SMA	DU145	23644382
PCA3	miR-1261	LNCaP	27743381
SChLAP1	miR-198	LNCaP PC3	28492138

Many miRs function as oncogenes or tumor suppressors in human cancers. Down-regulation of miR-145 has been reported in PC, suggesting that miR-145 functions as a tumor suppressor [69]. Clustering intersection analysis also linked miR-145 with PC. Significantly, He et al. found that miR-145 can regulate the expression of PCGEM1 by directly binding to target sites within the PCGEM1 sequence. PCGEM1 promote or suppress PC cell proliferation via miR-145 [25]. PCGEM1 and miR-145 exhibited reciprocal regulation: down-regulation of PCGEM1 expression in LNCaP cells increased expression of miR-145. Additionally, He et al. also found that both siRNA PCGEM1 and PmiR-145 transfection inhibited tumor cell proliferation, migration, invasion, and induced early apoptosis both in vitro [25]. Thus, it is obvious to find a reciprocal negative control relationship between PCGEM1 and miR-145 that regulates both LNCaP cell proliferation and PC tumor growth.

H19 is located at chromosome 11p15.5, and it was firstly discovered lncRNA in 1991, which is a member of a highly conserved cluster of imprinted genes [70]. It not only negatively regulates p53 protein and cell cycle progression or acts as a molecular sponge to regulate the let-7 family of microRNAs [65]. Previous study suggested that the H19-miR-675 signaling axis plays important roles in tumorigenesis [71]. It was later found that H19-miR-675 axis could suppress PC cell migration. In addition, Zhu et al. found that miR-675 potentially targets TGFBI. TGFBI (transforming growth factor β induced protein TGFBI) was an extra cellular matrix (ECM) protein involved in cancer metastasis. Conclusion, it is confirmed that the H19–miR-675 axis suppresses PC metastasis by down-regulation of TGFBI [65]. This is a novel understanding of the role of H19 and miR-675 in PC metastasis and the mechanism involved.

**Fig. 1:**
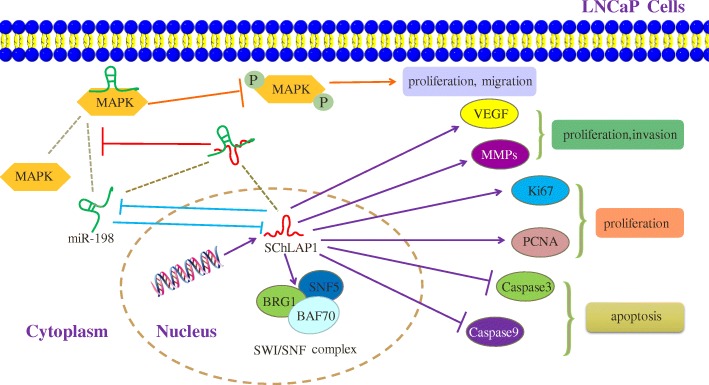
Regulate function of SChLAP1 in PC. Firstly, SChLAP1 and miR-198 can be suppressed to each other. The interaction of SChLAP1 and miR-198 represses the connection between MAPK1 and miR-198, thereby significantly inhibits the phosphorylation of MAPK1.SChLAP1 could promote PC cells migration and invasion byincreased the expression of VEGF, MMPs, Ki67 and PCNA. SChLAP1 could negatively regulated the expression of Caspase-3 and Caspase-9, thereby repress PC cells apoptosis. In addition, SChLAP1 can interact with the SWI/SNF-complex to resulte in cancer progression

Novel study showed that the expression of miR-198 is decreased in PC cells. According to bioinformatics analysis, there was a 18 bp matched sequence between miR-198 and SChLAP1, indicating that they may have a target specific selectivity. Further experiments showed that SChLAP1 and miR-198 can interact with each other. SChLAP1 negatively regulates the expression of miR-198. Similarly, over-expression of miR-198 partially inhibited SChLAP1. These results suggested that SChLAP1 may regulate the expression of miR-198 and subsequently influence the progression of PC. In addition, the interaction of SChLAP1 and miR-198 contribute to MAPK1 activation. MAPKs play important regulatory roles in cancer progression and MAPK1 has a potential binding domain with miR-198.SChLAP1 knockdown, which caused miR-198 upregulation, significantly inhibited the phosphorylation of MAPK1 [42]. Therefore, it suggested that targeting the SChLAP1-miR198-MAPK1 axis may represent a novel therapeutic application in PC (Fig. 1).

LncRNA PVT1 (plasma cytoma variant translocation 1), located at 8q24.21, is a powerful predictive factor of tumor progression and has been demonstrated to play important roles in various biological processes, such as proliferation, apoptosis, mobility and invasion [72, 73]. lncRNA PVT1 regulates PC cell growth by inducing the methylation of miR-146a. Firstly, miR-146a was significantly down-regulated in PC tissues, whereas the PVT1 expression was obviously upregulated. Linear regression analysis showed that the expression level of miR-146a was negatively correlated with the PVT1 in PC. Then the gain and loss-of-function analyses indicated that PVT1 knockdown caused a significant reduction in cell viability and a promotion in cell apoptosis. Finally, according to the MSP analysis, it provided evidences that PVT1 over-expression promoted the methylation of miR-146a CpG islands [74]. Taken together, these finding indicated that in PC, PVT1 exhibited oncogenic activity through the negative regulation of miRNA-146a by inducing the methylation in its promoter.

Xue et al. proved that miR-103 which is a member of the miR-103/107 family located on human chromosome 5, acts as a direct downstream target of lncRNA GAS5. It is consistently demonstrated that lncRNA GAS5 over-exposure resulted in significantly reduced phosphorylation of AKT and mTOR proteins, and further indicating that lncRNA GAS5 can lead to inactivate the AKT/mTOR signaling pathway [61]. AKT/mTOR signaling pathway plays a crucial role in various cellular functions, such as proliferation, growth, survival, and metabolism [75]. In addition, over-exposure of miR-103 could fully rescue the effect on the proteins related to the AKT/mTOR axis, including AKT and mTOR contributed by the up-regulation of lncRNA GAS5, which suggests that miR-103 might be a mediator for lncRNA GAS5 and the AKT/mTOR signaling pathway [61]. What counts is Li et al. found that miR-103 could function through activating the AKT/mTOR signaling pathway [76]. Therefore, it could be concluded that up-regulation of lncRNA GAS5 could inactivate the AKT/mTOR signaling pathway through targeting miR-103.

It had study revealed that ATF2 is a target gene of miR-204 [77]. Zhang et al. confirmed that miR-204 is a bonafide UCA1 targeting miRNA and lncRNA UCA1 could function as a ceRNA to promote ATF2 expression by sponging miR-204. Specifically, UCA1 directly interacted with miR-204 and decreased the binding of miR-204 to ATF2 3’UTR, which suppressed the degradation of ATF2 mRNA [64]. Furthermore, Fotouhi Ghiam A et al. found that UCA1 depletion induces radio sensitivity, decreases proliferative capacity and disrupts cell cycle progression in DU145 cells, it may occur through altered Akt signaling and induced cell cycle arrest at the G2/M transition [78]. Taken together, UCA1 could suppress PC cell progression through regulation of ATF2 by competitively binding miR-204 and the inhibition of UCA1 expression may be a promising strategy for PC therapy.

**Fig. 2:**
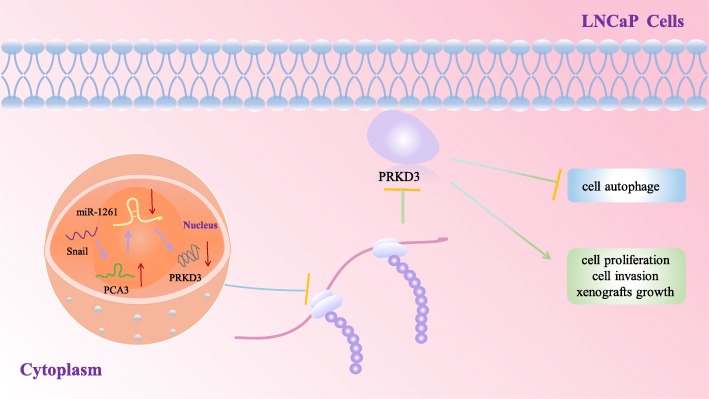
Regulate function of PCA3in PC. Firstly, PCA3expression can be activated by Snail. PCA3 could negatively regulate the expression of miRNA-1261. miR-1261 can bind to the PRKD3 and negatively regulated the expression of PRKD3, thereby promotes LNCaP cells proliferation, migration and invasion and inhibits cell autophage

PC antigen 3 (PCA3), also known as PC3DD3 or DD3PC3, is one such lncRNA that maps to chromosome 9q21–22 and is up-regulated in human PC. The up-regulated PCA3 promotes LNCaP cells proliferation, migration and invasion [79, 80]. It is reported that PCA3 is embedded within intron 6 of PRUNE2, a target protein-coding gene variant, and is transcribed in the antisense direction [81]. Ahmad Salameh et al. found expression or silencing of PRUNE2 in PC cells could decrease and increase cell proliferation, and they also confirmed that PCA3 downregulated PRUNE2 levels by binding its mRNA. The unique mechanism of PCA regulating PC involved formation of a PRUNE2/PCA3 double-stranded RNA that undergoes adenosine deaminase acting on RNA (ADAR)-dependent adenosine-to-inosine RNA editing. ADARs can bind PRUNE2/PCA3 dsRNA and regulate PRUNE2 Levels. Silencing ADAR1 increases PRUNE2 the levels in LNCaP cells and reduced PC cell proliferation in vitro and in vivo. Furthemore, Ahmad Salameh et al. also found intron6-PRUNE2 could also down-regulate PCA3. Thus, the PCA3/PRUNE2 axis plays a vital role in the development of in PC [80]. He et al. found that miR-1261 can bind to the PRKD3 3′ UTR which were at positions 2509–2515 and negatively regulated the expression of PRKD3 by affecting the transcription and translation of PRKD3 which contributes to PC cell growth and survival through a PKC epsilon/PKD3 pathway downstream of Akt and ERK [79]. However, there was one putative miR-1261 binding site in PCA3 (1715–1738 bp). Interestingly, PCA3 could negatively regulate the expression of miRNA-1261 in LNCaP cells. Moreover, He et al. analyzed the potential transfect factor binding sites in the promoter region of PCA3 and showed that there is one E-box element recognized by Snail. Taken together, Snail activated the expression of lncRNA PCA3 to inhibit the translation of PRKD3 protein via competitive miR-1261 sponging, and thus high expression of PRKD3 further promoted invasion and migration of PC. Since the PCA3 gene expressed only in prostate epithelial cells and has high tumor specificity [79]. Therefore, PCA3 has good clinical application value in the early diagnosis and treatment of PC (Fig. 2).

LncRNA HOXD-AS1, also known as HAGLR, is encoded in HOXD cluster and is evolutionary conserved among hominids and has all bona fide features of a gene [82]. HOXD-AS1 was over-expressed in androgen-independent PC-3 cells compared with LNCaP cells. The over-expressed expression of HOXD-AS1 enhanced the proliferation, castration resistance and chemo-resistance in LNCaP cells. Interestingly, knockdown of WDR5 abolished the effect of HOXD-AS1. Thus, these results suggest that HOXD-AS1 exerts its regulatory function in a WDR5-dependent mann. In addition, further investigation revealed that HOXD-AS1 recruited WDR5 to directly regulate the expression of target genes by mediating histone H3 lysine 4 tri-methylation (H3K4me3). According to a chromatin immunoprecipitation (ChIP) assay in control or HOXD-AS1 knockdown cells. HOXD-AS1 knockdown resulted in decreased location of WDR5, H3K4me3, and RNA polymerase-II levels in the promoter regions of PKL1, AURKA, FOXM1, CDC25C, UBE2C, CCNA2and CCNB1, but not in the negative control and BIRC5, suggesting that down-regulation of these genes was regulated directly by HOXD-AS1 [83]. Taken together, these data indicated that HOXD-AS1 activates the transcription of target genes directly in PC by recruiting WDR5 to mediate H3K4me3 at their promoter region.

**Fig. 3:**
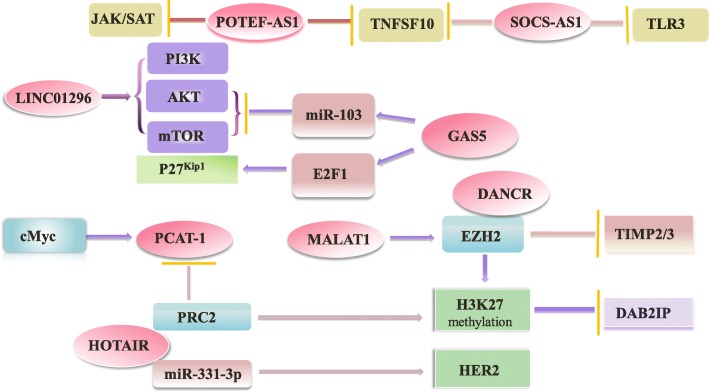
Overview of the role of lncRNAs in PC cells

**Fig. 4:**
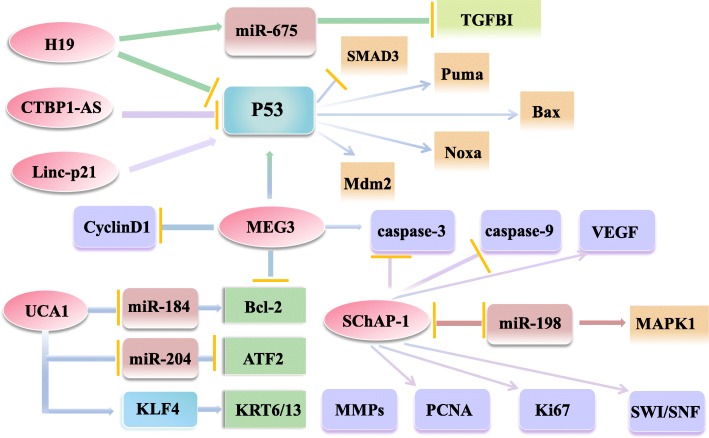
Overview of the role of lncRNAs in PC cells

In addition, according to the histopathological and clinicopathological analysis, MALAT1 could interacts with the N-terminal of zeste homolog 2 (EZH2) and regulates its methylating activity by GST pull-down and RIP assays to promotes PC progression [34]. HOTAIR can target HER2 mRNA by binding miR-331-3p, then modulates the depression of HER2 and imposes an additional level of post-transcriptional regulation thereby promoting the development of PC [84]. In sum, lncRNA can as decoys to bind and titrate away proteins or RNAs to impart specificity to genomic locations through either RNA-protein or RNA-miRNA recognition rules. Indirectly exert biological functions in multiple kingdoms of life (Figs. 3 and 4).

### Role of LncRNA in epithelial–mesenchymal transition

EMT was initially identified as a developmental process from an epithelial phenotype to an invasive mesenchymal phenotype. Many transcriptional factors including Twist, Snail, Slug and Zeb1/2 are involved in this process [85]. EMT also plays essential roles in cancer cell invasion and metastasis [85]. In PC, elevated levels of mesenchymal biomarkers and reduced epithelial differentiation markers are highly correlated with invasion and metastasis.

LncRNA-ATB (activated by TGF-β), located on chromosome 14, was first reported as highly expressed in hepatocellular carcinoma (HCC) and showed extensive regulatory functions [86]. The lncRNA-ATB expressed in 57 pairs of PC and adjacent non-cancerous prostate tissues. The expression of lncRNA-ATB was significantly increased in the PC patients. Knockdown of lncRNA-ATB inhibited the growth of PC cells via regulations of cell cycle regulatory protein expression levels. Knockdown of lncRNA-ATB significantly rescued the increased expression levels of epithelial cells (E-cadherin and ZO-1), which have the function of ell–cell adhesion and polarity and decreased expression levels of mesenchymal cells(N-cadherin and Vimentin), which have the function of migratory, invasive and anti-apoptotic potential in PC-3 and DU-145 prostate cell line [2]. Amusingly, Colon cancer-associated transcript 2 (CCAT2), which mapping to 8q24, has the similar mechanism inhibiting PC cells growth with lncRNA-ATB [87]. So it is proposed that lncRNA-ATB and CCAT-2 promotes the PC migration and invasion partly via the induction of EMT. In addition, up-regulation of lncRNA-ATB promoted the expression levels of ZEB1 and ZNF217 at mRNA and protein levels in PC-3 and DU-145 prostate cell lines. These results indicated that lncRNA-ATB may drive EMT in PC cells via positive modulation of ZEB1 and ZNF217 expression levels [2].

Both MAPK/ERK pathways and PI3K-Akt pathways are essential intracellular signaling pathways involving in the proliferation and EMT during the processes of cancers [88]. According to the results of Xu et al.’ experiment, it indicated that both PI3K/Akt and ERK signaling pathways involved lncRNA-ATB-mediated growth and EMT of human PC. Consequently, proliferation rate of PC cells is enhanced upon over-expression of lncRNA-ATB [2]. Taken together, lncRNA-ATB might be considered as a novel molecular target for the diagnosis and treatment of PC.

LINC01296 knockdown significantly suppressed the EMT process by reducing the expression levels of N-cadherin, vimentin and MMP9, and by increasing E-cadherin expression compared with the control group. MMP9, a gene that is usually upregulated during EMT in cancer cells [4]. Thus, it mechanistically demonstrated that LINC01296 is possibly an inducing factor of EMT, making it an important factor in PC metastasis.

### Potential clinical application of lncRNAs in PC

Whole genome transcriptomic analyses have identified numerous lncRNA transcripts that are increasingly implicated in cancer biology. LncRNAs are found to promote essential cancer cell functions such as proliferation, invasion and metastasis, with the potential to serve as novel biomarkers of various cancers and to further reveal uncharacterized aspects of tumor biology. However, the biological and molecular mechanisms as well as the clinical applications of lncRNAs in diverse diseases are not completely understood and remain to be fully explored. LncRNAs may be critical players and regulators in PC carcinogenesis and progression, and could serve as potential biomarkers for PC [33]. The ideal and convenient biomarkers should possess several typical and important characteristics. First of all, it should be available for access and collected by means of biopsy or surgical resection. Second, it should be detected easily in body fluids such as blood, urine and semen, and its expression is significantly different between PC and normal tissues or cells. More importantly, its abnormal expression is significantly associated with patients’ clinical features such as PSA, Gleason score and metastasis. Therefore, the goal of this section was to sum up most of reported lncRNAs which could be used as diagnostic and prognostic biomarkers for PC. (Table 3).

**Table 3:** Application index of lncRNAs in PC

LncRNA	Cutoff value	AUC	Sensitivity	Specificity	Reference(PMID)
MALAT1		0.860,0.790	0.586	0.848	28272371
PCAT-14		0.837,0.823			27566105
LincRNA-p21		0.663	0.670	0.630	25999983
LincRNA-ATB	1.3	0.931	1.000	0.833	27176634
LincRNA-FR0348383		0.599	0.645	0.815	25597901
SChLAP1		0.660			25499224

### lncRNA as diagnostic marker

Ren et al. have reported that the expression of MALAT1 in human PC tissues and cell lines was closely associated with high PSA levels, Gleason scores, and tumor sizes [89]. Wang et al. have shown that the use of a MALAT1 model, which may serve as an independent predictor of PC, would prevent unnecessary biopsies in about 30.2–46.5% of patients with serum PSA levels in the “diagnostic grey zone” (PSA 4–10 ng/mL) [90]. Moreover, Ren et al. have found that MALAT-1 derived miniRNA (MD-miniRNA) from plasma may be used as a novel approach to detect human PC. Compared to non-PC patients, the plasma MD-miniRNA levels are significantly elevated in PC patients (*p* < 0.001). At a cut-off of 867.8 copies/mL of plasma MD-miniRNA, the sensitivity and specificity for distinguishing PC from non-PC was 58.6 and 84.8%, respectively, and the sensitivity and specificity for distinguishing positive from negative biopsies was 43.5 and 81.6%, respectively [91]. Similarly, Xue et al. have demonstrated that MD-miniRNA had a relatively high diagnostic accuracy with an AUC of 0.86 (95% CI 0.80–0.93) and 0.79 (95% CI 0.70–0.88) to discriminate PC patients from healthy controls and PC patients from BPH patients, respectively. Meanwhile, a combination of PSA and MD-miniRNA showed a better diagnostic performance compared to either single biomarker [92]. Additionally, as an independent PC predictor (*p* < 0.001), the urinary FR0348383 score, defined as the ratio of PSA mRNA and FR0348383 level (PSA mRNA/FR0348383 lncRNA × 1000), shows a much more outstanding performance than PSA and its derivates, including %free PSA and PSA density (AUC: 0.815 vs. 0.562, 0.599, and 0.645, respectively) in the subgroup of patients with a PSA value in the grey area [93]. In sum, these data indicate that MALAT1 and FR0348383 are useful diagnostic and predictive biomarker for PC detection and warrants further study in clinical trials with a large sample size.

### lncRNA as prognostic marker

Expression of lncRNA-ATB was significantly increased in the PC patients in comparison with the adjacent noncancerous prostate tissues. The diagnostic efficacy of lncRNA-ATB in PC tissues of PC patients was evaluated by calculating the area under the receiver operating characteristic curve. The ROC curve analysis revealed that AUC was 0.931. When the cutoff value = 1.30, the diagnostic sensitivity (100%) and specificity (83.3%) reached their peak values (*P* < 0.05). Thus the lncRNA ATB expression was further classified into the low expression group (n = lncRNA-ATB expression < 1.30, *n* = 32) and high expression group (lncRNA-ATB expression ≥1.30, *n* = 25) as the threshold ROC curve value of 1.30. In addition, the high lncRNA-ATB expression in PC tissues of PC patients is closely related with aggressive clinical pathological parameters including histological grade (*P* = 0.017), high preoperative PSA level (*P* = 0.001), pathological stage (*P* = 0.003), high Gleason score (*P* = 0.045), lymph node metastasis (*P* = 0.007), angiolymphatic invasion (*P* = 0.008), biochemical recurrence (*P* = 0.003). However, there was no correlation between lncRNA-ATB with age (*P* = 0.952) and surgical margin status (*P* = 0.257). The Cox multivariate analysis was conducted to assess the relationship of lncRNA-ATB expression with the BCR-free survival of patients with PC. The results established the significance of lncRNA-ATB expression (*P* < 0.001), and other clinicopathological parameters including histological grade (*P* = 0.020), preoperative PSA level (*P* = 0.002), Gleason score (*P* = 0.049), pathological stage ( < 0.001), lymph node metastasis (*P* = 0.025) and angiolymphatic invasion (*P* = 0.011) for independent prognostic predictors of BCR-free survival of PC patients [2]. Similar to lncRNA-ATB, LINC01296, located at chromosome 14q11.2, has the same clinicopathologic features and patients with higher LINC01296 expression displayed advanced clinical features and shorter biochemical recurrence-free survival time than those with lower LINC01296 expression [4]. Taken together, lncRNA-ATB and LINC01296may be considered as a new predictor in the clinical prognosis and therapeutic target of patients with PC.

The gene encoding lnc-MX1–1 is located in chromosome 21and the mature lnc-MX1–1 transcript is of 1182 nt in length which was sequenced by high throughput RNA-seq analysis [94]. lnc-MX1–1 is over-expressed in PC tissues compared with their adjacent normal prostate tissues by gene expression array profiling. Moreover, Jiang et al. confirmed that there was a significant association between over-expression of lnc-MX1–1 and patients’ clinical features such as PSA, Gleason score, metastasis, and recurrence free survival. The expression level of lnc-MX1–1 was significantly different among PSA stratification and increased along with PSA values (*P* = 0.0038). The expression of lnc-MX1–1 was remarkably higher (*P* < 0.001) in patients with high Gleason score (> 7) or in which bone or lymph node metastases were present (*P* = 0.0126) compared to earlier tumor stages. The recurrence free survival was significantly lower in patients with high lnc-MX1–1 expression level compared to those with low expression level (*P* = 0.0262) [95]. In conclusion, these results suggest that lnc-MX1–1 may serve as a potential prognostic biomarker and therapeutic target for PC.

PCAT-14 (PC associated transcript-14) is a polyatomic gene found within a gene desert on chromosome 22, with a striking PC and lineage specific expression pattern across the N10,000 TCGA cancer and normal tissue samples [96]. White et al. performed an integrative analysis and found androgen-regulated PCAT-14 as the most prevalent lncRNA that was over-expressed in prostate tumors relative to normal prostate. Lower PCAT-14 expression was associated with increasing Gleason score and poor outcome [97]. Meanwhile, Shukla et al. demonstrated that expression of PCAT-14 is prognostic of outcome and is associated with better biochemical progression-free survival, metastases-free survival, and PC-specific survival. As the univariate analysis showed patients with high PCAT-14 expression were significantly associated with better BPFS, MFS, PSS and OS. In addition, in both the TCGA and Taylor PC cohorts, PCAT-14 expression was able to significantly distinguish cancer from normal with an AUC of 0.837 and 0.823 respectively supporting its utility as a diagnostic biomarker [96]. Similarly, SChLAP1, UCA1 and CCAT2 are all upregulated in PC, but patients with higher expression level of them had poorer overall survival and progression-free survival [78, 87, 98]. To sum up, PCT14, SChLAP1, UCA1 and CCAT2 might play a crucial role in human PC and serve as a new potential biomarker for clinical prognosis evaluation.

Another study investigated the lincRNA-p21 level in PC exosomes. An ROC curve was constructed for differentiating PC patients from controls. Compared to matched adjacent nontumorous tissues, the specificity of lincRNA-p21 for PC was 94% when combined with PSA, the AUC was up to (AUC: 0.663, CI:95%) and the sensitivity and specificity were 67 and 63%. In addition, the correlation between lincRNA-p21 level and clinical parameters was analyzed by chi-square test and Fisher exact test. The results showed that low lincRNA-p21 expression was associated with high tumor stage, Gleason grade and PS. Moreover, analyzing the correlation between lincRNA-p21 level and patients’ survival, the survival analysis showed that low lincRNA-p21 level predicts both overall and disease-free survival [52, 54]. It is identical to GAS5 and HCG11, which located on chromosome 6q22.2 and was down-regulated in tumor tissues compared with non-tumor tissues in both databases [99]. Taken together, lincRNA-p21, HCG11 and GAS5 might be a candidate biomarker for prognosis evaluation.

Delayed diagnosis, recurrence, and metastasis are the biggest obstacles to the treatment of PC. Therefore, searching for the ideal biomarkers of PC is essential for improving the early diagnostic rate. The findings above indicate that lncRNA (FR0348383, MALAT1, PCA3, SChLAP1, PCAT-14, LincRNA-p21, HCG11, lncRNA-ATB) might be used as a potential marker for PC diagnosis. However, the precise molecular mechanisms by which lncRNA functions in PC remain obscure. Further exploratory and validation research is needed to elucidate the functional role of lncRNA in PC, especially in clinical applications.

### Future expectations

After the discovery of ncRNA such as lncRNA and microRNA, increasing researches report dysregulated lncRNA expression among various cancers and suggest that lncRNA is closely related to tumorigenesis, metastasis or recurrence either as oncogenes or tumor suppressors. Furthermore, lncRNAs may serve as independent biomarkers for cancer diagnosis and prognosis. Nevertheless, biological information about lncRNAs is far from adequate compared to that of protein-coding genes. The major hurdle for functional research of lncRNAs is the lack of effective tools to inhibit their transcription. Many lncRNAs are localized in the nucleus, difficult to be knocked down by RNAi [100, 101]. Thus, additional studies about lncRNA should be done for defining.

Firstly, in recent research, Zhen et al. confirmed that CRISPR/Cas9 can inhibit the expression of long non-coding RNA UCA1, which acted as an oncogenic lncRNA in bladder cancer, to attenuate malignant phenotypes of bladder cancer [102]. CRISPR (Clustered regularly interspaced short palindromic repeats) is identified as a natural bacterial immunity system, promising a cheap, simple and versatile genome editing technique [103]. In view of its specificity, efficiency, simplicity and versatility, the CRISPR/Cas9 technique has achieved numerous successes as a robust genome engineering tool for the treatment of many diseases [102]. It is worth noting that the lncRNA UCA1 is also up-regulated in PC and it promotes PC cell proliferation, migration and invasion [64]. These results suggest that additional studies are required. Could CRISPR/Cas9 be used to treat PC by inhibiting the expression of UCA1 in PC tissues? If so, what is the molecular mechanism of action?

Secondly, long intragenic non-coding RNA lincRNA-p21 is found in exosomes by Isin et al. [52]. In addition, Wang et al. found that lincRNA-p21 level was significantly down-regulated in PC tissues compared with normal prostate tissues. The down-regulated lincRNA-p21 can promote PC cells proliferation [54]. Interestingly, Pan et al. demonstrated that the newly identified long noncoding RNA ZFAS1 in GC (gastric carcinoma), which was enriched in exosomes from the serum of GC patients, could be transmitted by exosomes to enhance GC cell proliferation and migration. Moreover, Pan et al. also confirmed that ZFAS1 may be used as a potential biomarker for GC diagnosis and a novel target for GC therapy [104]. Thus, if we can look for ZFAS1 in the exosomes of PC patients and use it as a diagnostic biomarker and a novel target for PC patients therapy, this filed is worthy of widely attention.

## Conclusion

A number of genome’s repertoire of non-protein-coding transcripts may be viewed as inconsequential transcriptional “noise” “garbage” [105]. However, with the high-throughput sequencing technologies (such as microarray or RNA-seq) progression, something important hidden in the multifarious ncRNAs has drawn the attention of many scientists in their search for the pathogenesis of diseases [106, 107]. More importantly, the lncRNA study has gradually become one of the most noticeable parts in the field of RNA biology. It is becoming clear that lncRNA possess critical biological functions in PC. High-throughput sequencing technologies have revealed numerous lncRNA transcripts that play pivotal roles in PC. Thus, they play under appreciated but critical roles in normal cell dynamics. In this review, we highlighted the dysregulation of lncRNA in PC and concluded that lncRNAs are important regulators in PC biology and may be potential therapeutic targets. However, only a few lncRNAs have been well characterized in PC. As the new research field of lncRNA is expanding quickly, it is meaningful to elucidate the possible functions and underlying regulatory pathway of the aberrant expression of lncRNAs in PC. Although accumulating evidence supports epigenetic biomarkers and the therapeutic potential of lncRNAs for the aforementioned diseases, there are lots of unresolved questions about their regulation. However, the exact function of many lncRNAs is still unknown, since they do not necessarily have only one target or function within a cell. In addition, the same lncRNA may exert different functions depending on the type of tumor. Therefore, using lncRNAs as therapeutic targets may entail unforeseeable side effects or dramatic adverse reactions. Nevertheless, the better the function of lncRNAs is understood, the more efficient and broader their field of therapeutic usage will be. Further study performed in vitro in cells and in vivo in rodents with advanced techniques, such as microarray and next-generation sequencing, will unravel the mystery of lncRNA regulation in these disorders. Taken together, integration of functional lncRNA biology into PC biology may further deepen our understanding of the mechanisms of PC and then some specific lncRNAs have the potential to be translated into clinical applications for diagnosis, prognosis or treatment of PC.

## Abbreviations

AR: Androgen Receptor
Bcl-2: B-cell lymphoma-like 2
CCAT2: Colon cancer-associated transcript 2
ChIP: Chromatin immunoprecipitation
CR: Castration resistant
CRISPR: Clustered regularly interspaced short palindromic repeats
DANCR: Differentiation antagonising non-protein coding RNA
DRAIC: Down-regulated RNA in androgen independent cells
ECM: Extra cellular matrix
EMT: Epithelial-mesenchymal transition
ENCODE: Encyclopedia of DNA elements
EZH2: Enhancer of zeste homolog 2
FOXA1: the transcription factors forkhead box protein A1
GC: Gastric carcinoma
GENCODE: Encyclopædia of genes and gene variants
H3K27me3: Histone 3 lysine 27 trimethylation
H3K4me3: mediating histone H3 lysine 4 tri-methylation
HCC: Hepatocellular carcinoma
HDAC: Histone Decarboxylase
hnRNP-K: Heterogeneous nuclear ribonucleoprotein K
HOTAIR: HOX transcription antisense RNA
lncRNA PVT1: plasma cytoma variant translocation 1
lncRNA SChLAP1: second chromosome locus associated with prostate-1
lncRNAs: Long non-coding RNAs
LSD1: Lysine Specific Demethylase 1
MALAT-1: Metastasis-associated lung adenocarcinoma transcript 1
MEG3: Maternally Expressed Gene 3
MMPs: Matrix metalloproteinases
NKX3–1: homeobox proteinNKX3–1
PC: Prostate cancer
PC3: Prostate cancer antigen 3
PCAT1: PC-associated intergenic non-coding RNA transcript 1
PCAT14: PC-associated transcript-14
PCAT29: PC-associated transcript 29
PCGEM1: PC gene expression marker 1
PRC2: Polycomb repressive complex 2
SMAD3: Mothers against decapentaplegic homolog 3
SOCS2-AS1: The lncRNA Suppressor of cytokine signalling 2-antisense transcript 1
TGFBI: Transforming growth factor β induced protein TGFBI
TSS: Transcription start site
UCA1: Human urothelial carcinoma associated 1
VEGF: Vascular endothelial growth factor
